# Accurate prediction of protein structures and interactions using a 3-track neural network

**DOI:** 10.1126/science.abj8754

**Published:** 2021-07-15

**Authors:** Minkyung Baek, Frank DiMaio, Ivan Anishchenko, Justas Dauparas, Sergey Ovchinnikov, Gyu Rie Lee, Jue Wang, Qian Cong, Lisa N. Kinch, R. Dustin Schaeffer, Claudia Millán, Hahnbeom Park, Carson Adams, Caleb R. Glassman, Andy DeGiovanni, Jose H. Pereira, Andria V. Rodrigues, Alberdina A. van Dijk, Ana C. Ebrecht, Diederik J. Opperman, Theo Sagmeister, Christoph Buhlheller, Tea Pavkov-Keller, Manoj K Rathinaswamy, Udit Dalwadi, Calvin K Yip, John E Burke, K. Christopher Garcia, Nick V. Grishin, Paul D. Adams, Randy J. Read, David Baker

**Affiliations:** 1 Department of Biochemistry, University of Washington; Seattle, WA98195, USA; 2 Institute for Protein Design, University of Washington; Seattle, WA98195, USA; 3 Faculty of Arts and Sciences, Division of Science, Harvard University; Cambridge, MA02138, USA; 4 John Harvard Distinguished Science Fellowship Program, Harvard University; Cambridge, MA 02138, USA; 5 Eugene McDermott Center for Human Growth and Development, University of Texas Southwestern Medical Center; Dallas, TX, USA; 6 Department of Biophysics, University of Texas Southwestern Medical Center; Dallas, TX, USA; 7 Department of Biochemistry, University of Texas Southwestern Medical Center; Dallas, TX, USA; 8 Howard Hughes Medical Institute, University of Texas Southwestern Medical Center; Dallas, TX, USA; 9 Department of Haematology, Cambridge Institute for Medical Research, University of Cambridge; Cambridge, U.K; 10 Program in Immunology, Stanford University School of Medicine, Stanford, CA 94305, USA; 11 Departments of Molecular and Cellular Physiology and Structural Biology, Stanford University School of Medicine, Stanford, CA 94305, USA; 12 Molecular Biophysics & Integrated Bioimaging Division, Lawrence Berkeley National Laboratory, Berkeley, CA, USA; 13 Department of Biochemistry, Focus Area Human Metabolomics, North-West University; 2531 Potchefstroom, South Africa; 14 Department of Biotechnology, University of the Free State; 205 Nelson Mandela Drive, Bloemfontein, 9300, South Africa; 15 Institute of Molecular Biosciences, University of Graz; Humboldtstrasse 50, 8010, Graz, Austria; 16 Medical University of Graz; Graz, Austria; 17 BioTechMed-Graz; Graz, Austria; 18 Department of Biochemistry and Microbiology, University of Victoria; Victoria, British Columbia, Canada; 19 Life Sciences Institute, Department of Biochemistry and Molecular Biology, The University of British Columbia; Vancouver, British Columbia, Canada; 20 Howard Hughes Medical Institute, Stanford University School of Medicine, Stanford, CA 94305, USA; 21 Department of Bioengineering, University of California Berkeley, Berkeley, CA 94720, USA; 22 Howard Hughes Medical Institute, University of Washington; Seattle, WA98195, USA

## Abstract

DeepMind presented remarkably accurate predictions at the recent CASP14 protein structure prediction assessment conference. We explored network architectures incorporating related ideas and obtained the best performance with a 3-track network in which information at the 1D sequence level, the 2D distance map level, and the 3D coordinate level is successively transformed and integrated. The 3-track network produces structure predictions with accuracies approaching those of DeepMind in CASP14, enables the rapid solution of challenging X-ray crystallography and cryo-EM structure modeling problems, and provides insights into the functions of proteins of currently unknown structure. The network also enables rapid generation of accurate protein-protein complex models from sequence information alone, short circuiting traditional approaches which require modeling of individual subunits followed by docking. We make the method available to the scientific community to speed biological research.

The prediction of protein structure from amino acid sequence information alone has been a longstanding challenge. The bi-annual Critical Assessment of Structure (CASP) meetings have demonstrated that deep learning methods such as AlphaFold (1, 2) and trRosetta (3), that extract information from the large database of known protein structures in the PDB, outperform more traditional approaches that explicitly model the folding process. The outstanding performance of DeepMind’s AlphaFold2 in the recent CASP14 meeting (https://predictioncenter.org/casp14/zscores_final.cgi) left the scientific community eager to learn details beyond the overall framework presented and raised the question of whether such accuracy could be achieved outside of a world-leading deep learning company. As described at the CASP14 conference, the AlphaFold2 methodological advances included 1) starting from multiple sequence alignments (MSAs) rather than from more processed features such as inverse covariance matrices derived from MSAs, 2) replacement of 2D convolution with an attention mechanism that better represents interactions between residues distant along the sequence, 3) use of a two-track network architecture in which information at the 1D sequence level and the 2D distance map level is iteratively transformed and passed back and forth, 4) use of an SE(3)-equivariant Transformer network to directly refine atomic coordinates (rather than 2D distance maps as in previous approaches) generated from the two-track network, and 5) end-to-end learning in which all network parameters are optimized by backpropagation from the final generated 3D coordinates through all network layers back to the input sequence.

## Network architecture development

**Fig. 1:**
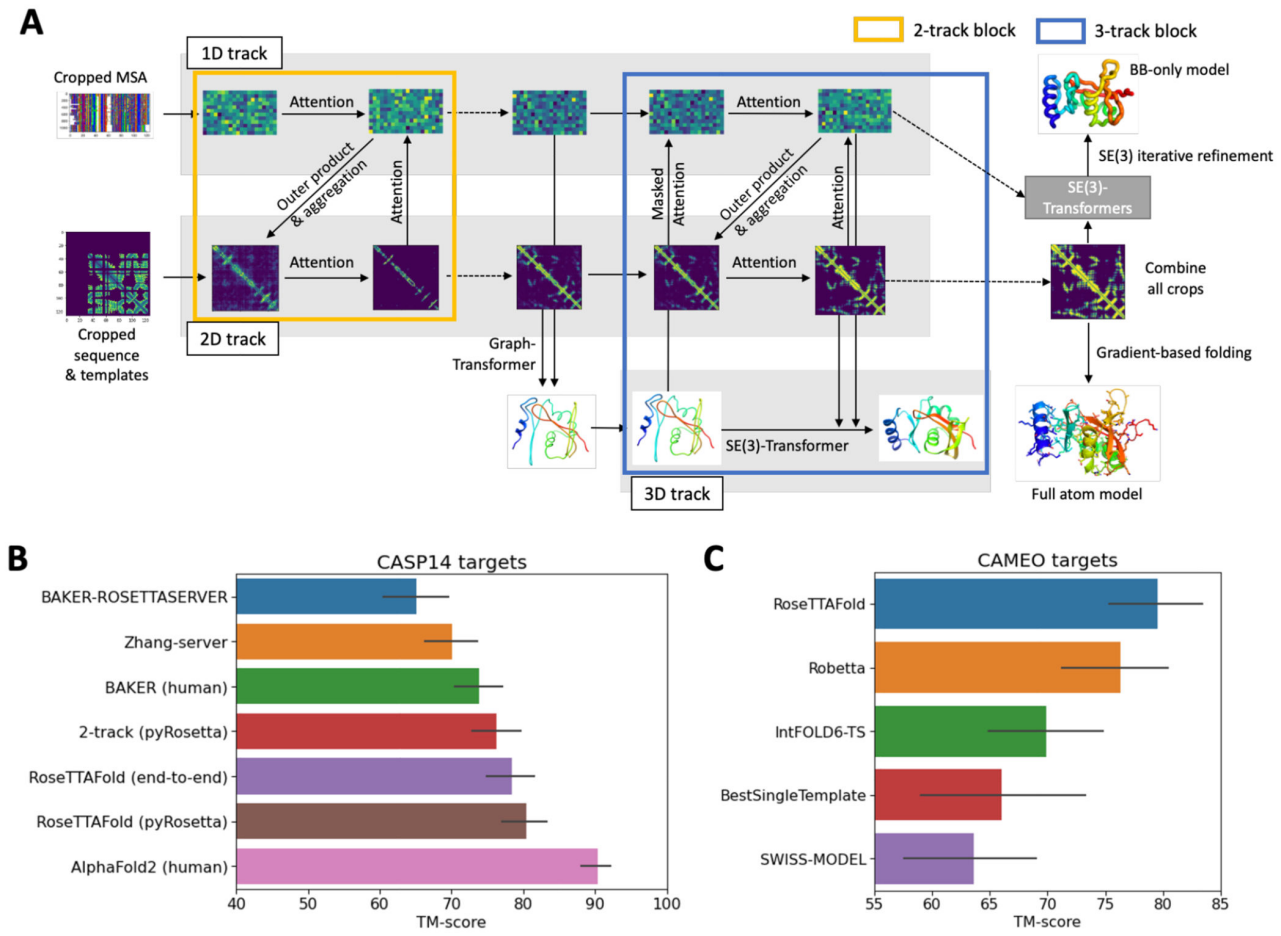
Network architecture and performance. (A) RoseTTAFold architecture with 1D, 2D, and 3D attention tracks. Multiple connections between tracks allow the network to simultaneously learn relationships within and between sequences, distances, and coordinates (see Methods and fig. S1 for details). (B) Average TM-score of prediction methods on the CASP14 targets. Zhang-server and BAKER-ROSETTASERVER were the top 2 server groups while AlphaFold2 and BAKER were the top 2 human groups in CASP14; BAKER-ROSETTASERVER and BAKER predictions were based on trRosetta. Predictions with the 2-track model and RoseTTAFold (both end-to-end and pyRosetta version) were completely automated. (C) Blind benchmark results on CAMEO medium and hard targets; model accuracies are TM-score values from the CAMEO website (https://cameo3d.org/).

Intrigued by the DeepMind results, and with the goal of increasing protein structure prediction accuracy for structural biology research and advancing protein design (4), we explored network architectures incorporating different combinations of these five properties. In the absence of a published method, we experimented with a wide variety of approaches for passing information between different parts of the networks, as summarized in the Methods and table S1. We succeeded in producing a “two-track” network with information flowing in parallel along a 1D sequence alignment track and a 2D distance matrix track with considerably better performance than trRosetta (BAKER-ROSETTASERVER and BAKER in Fig. 1B), the next best method after AlphaFold2 in CASP14 (https://predictioncenter.org/casp14/zscores_final.cgi).

We reasoned that better performance could be achieved by extending to a third track operating in 3D coordinate space to provide a tighter connection between sequence, residue-residue distances and orientations, and atomic coordinates. We constructed architectures with the two levels of the two-track model augmented with a third parallel structure track operating on 3D backbone coordinates as depicted in Fig. 1A (see Methods and fig. S1 for details). In this architecture, information flows back and forth between the 1D amino acid sequence information, the 2D distance map, and the 3D coordinates, allowing the network to collectively reason about relationships within and between sequences, distances, and coordinates. In contrast, reasoning about 3D atomic coordinates in the two-track AlphaFold2 architecture happens after processing of the 1D and 2D information is complete (although end-to-end training does link parameters to some extent). Because of computer hardware memory limitations, we could not train models on large proteins directly as the 3-track models have many millions of parameters; instead, we presented to the network many discontinuous crops of the input sequence consisting of two discontinuous sequence segments spanning a total of 260 residues. To generate final models, we combined and averaged the 1D features and 2D distance and orientation predictions produced for each of the crops and then used two approaches to generate final 3D structures. In the first, the predicted residue-residue distance and orientation distributions are fed into pyRosetta (5) to generate all-atom models. In the second, the averaged 1D and 2D features are fed into a final SE(3)-equivariant layer (6), and following end-to-end training from amino acid sequence to 3D coordinates, backbone coordinates are generated directly by the network (see Methods). We refer to these networks, which also generate per residue accuracy predictions, as RoseTTAFold. The first has the advantage of requiring lower memory (for proteins over 400 residues, 8GB rather than 24GB) GPUs at inference time and producing full side chain models, but requires CPU time for the pyRosetta structure modeling step.

The 3-track models with attention operating at the 1D, 2D, and 3D levels and information flowing between the three levels were the best models we tested (Fig. 1B), clearly outperforming the top 2 server groups (Zhang-server and BAKER-ROSETTASERVER), BAKER human group (ranked second among all groups), and our 2-track attention models on CASP14 targets. As in the case of AlphaFold2, the correlation between multiple sequence alignment depth and model accuracy is lower for RoseTTAFold than for trRosetta and other methods tested at CASP14 (fig. S2). The performance of the 3-track model on the CASP14 targets was still not as good as AlphaFold2 (Fig. 1B). This could reflect hardware limitations that limited the size of the models we could explore, alternative architectures or loss formulations, or more intensive use of the network for inference. DeepMind reported using several GPUs for days to make individual predictions, whereas our predictions are made in a single pass through the network in the same manner that would be used for a server; following sequence and template search (~1.5 hours), the end-to-end version of RoseTTAFold requires ~10 minutes on an RTX2080 GPU to generate backbone coordinates for proteins with less than 400 residues, and the pyRosetta version requires 5 minutes for network calculations on a single RTX2080 GPU and an hour for all-atom structure generation with 15 CPU cores. Incomplete optimization due to computer memory limitations and neglect of side chain information likely explain the poorer performance of the end-to-end version compared to the pyRosetta version (Fig. 1B; the latter incorporates side chain information at the all-atom relaxation stage); since SE(3)-equivariant layers are used in the main body of the 3-track model, the added gain from the final SE(3) layer is likely less than in the AlphaFold2 case. We expect the end-to-end approach to ultimately be at least as accurate once the computer hardware limitations are overcome, and side chains are incorporated.

The improved performance of the 3-track models over the 2-track model with identical training sets, similar attention-based architectures for the 1D and 2D tracks, and similar operations in inference (prediction) mode suggests that simultaneously reasoning at the multiple sequence alignment, distance map, and three-dimensional coordinate representations can more effectively extract sequence-structure relationships than reasoning over only MSA and distance map information. The relatively low compute cost makes it straightforward to incorporate the methods in a public server and predict structures for large sets of proteins, for example, all human GPCRs, as described below.

Blind structure prediction tests are needed to assess any new protein structure prediction method, but CASP is held only once every two years. Fortunately, the Continuous Automated Model Evaluation (CAMEO) experiment (7) tests structure prediction servers blindly on protein structures as they are submitted to the PDB. RoseTTAFold has been evaluated since May 15th, 2021 on CAMEO; over the 69 medium and hard targets released during this time (May 15th, 2021 ~ June 19th, 2021), it outperformed all other servers evaluated in the experiment including Robetta (3), IntFold6-TS (8), BestSingleTemplate (9), and SWISS-MODEL (10) (Fig. 1C).

We experimented with approaches for further improving accuracy by more intensive use of the network during sampling. Since the network can take as input templates of known structures, we experimented with a further coupling of 3D structural information and 1D sequence information by iteratively feeding the predicted structures back into the network as templates and random subsampling from the multiple sequence alignments to sample a broader range of models. These approaches generated ensembles containing higher accuracy models, but the accuracy predictor was not able to consistently identify models better than those generated by the rapid single pass method (fig. S3). Nevertheless, we suspect that these approaches can improve model performance and are carrying out further investigations along these lines.

In developing RoseTTAFold, we found that combining predictions from multiple discontinuous crops generated more accurate structures than predicting the entire structure at once (fig. S4A). We hypothesized that this arises from selecting the most relevant sequences for each region from the very large number of aligned sequences often available (fig. S4B). To enable the network to focus on the most relevant sequence information for each region while keeping access to the full multiple sequence alignment in a more memory efficient way, we experimented with the Perceiver architecture (11), updating smaller seed MSAs (up to 100 sequences) with extra sequences (thousands of sequences) through cross-attention (fig. S4C). Current RoseTTAFold only uses the top 1000 sequences due to memory limitations; with this addition, all available sequence information can be used (often over 10,000 sequences). Initial results are promising (fig. S4D), but more training will be required for rigorous comparison.

## Enabling experimental protein structure determination

With the recent considerable progress in protein structure prediction, a key question is what accurate protein structure models can be used for. We investigated the utility of the RoseTTAFold to facilitate experimental structure determination by X-ray crystallography and cryo-electron microscopy and to build models providing biological insights for key proteins of currently unknown structures.

**Fig. 2:**
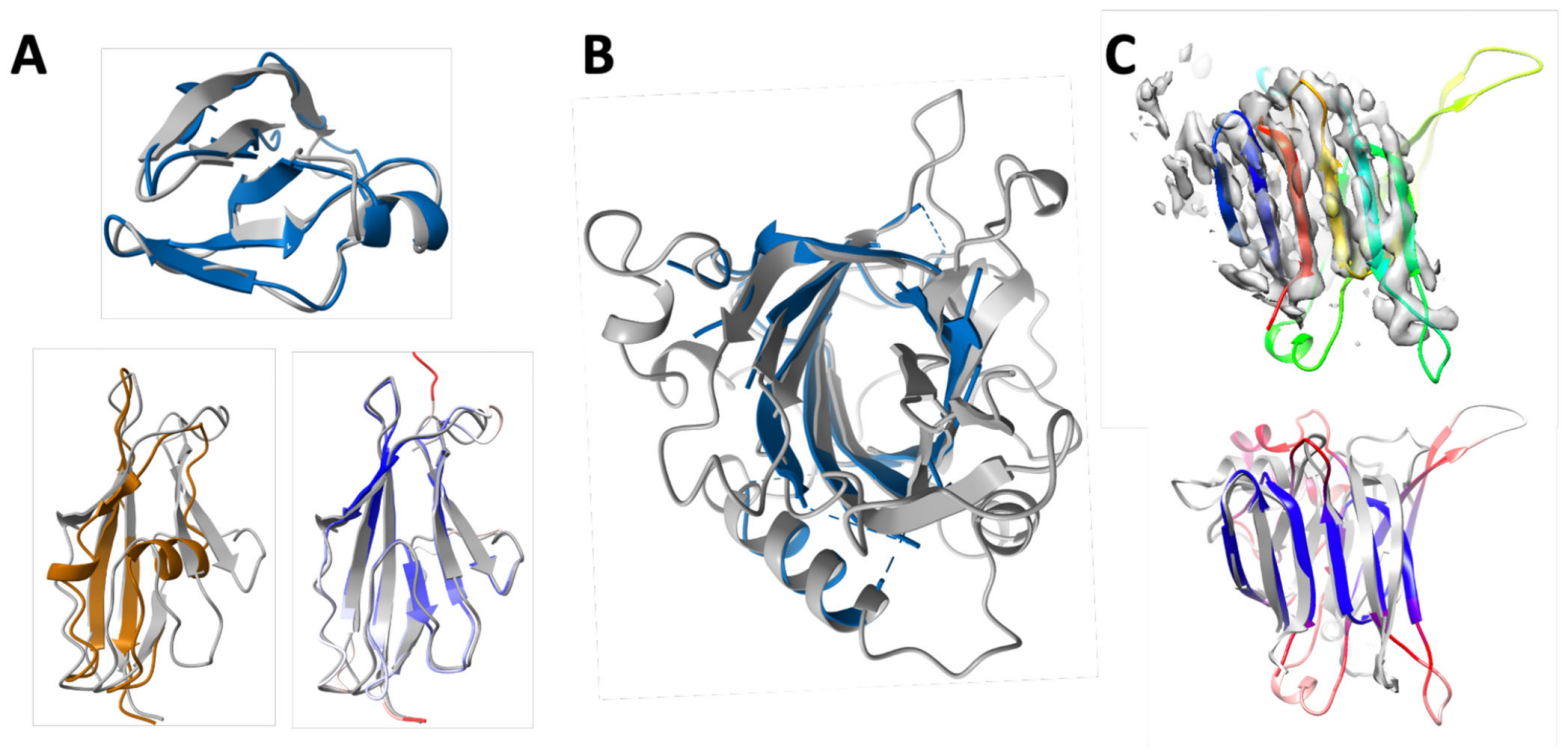
Enabling experimental structure determination with RoseTTAFold. **(A-B)** Successful molecular replacement with RoseTTAFold models. **(A)** SLP. (top) C-terminal domain: comparison of final refined structure (gray) to RoseTTAFold model (blue); there are no homologs with known structure. (bottom) N-terminal domain: refined structure is in gray, and RoseTTAFold model is colored by the estimated RMS error (ranging from blue for 0.67 Å to red for 2 Å or greater). 95 Cα atoms of the RoseTTAFold model can be superimposed within 3 Å of Cα atoms in the final structure, yielding a Cα-RMSD of 0.98 Å. In contrast, only 54 Cα atoms of the closest template (4l3a, brown) can be superimposed (with a Cα-RMSD of 1.69 Å). **(B)** Refined structure of Lrbp (gray) with the closest RoseTTAFold model (blue) superimposed; residues having estimated RMS error greater than 1.3 Å are omitted (full model is in fig. S5C). **(C)** Cryo-EM structure determination of p101 Gβγ binding domain (GBD) in a heterodimeric PI3Kγ complex using RoseTTAFold. (top) RoseTTAFold models colored in a rainbow from the N-terminus (blue) to the C-terminus (red) have a consistent all-beta topology with a clear correspondence to the density map. (bottom) Comparison of the final refined structure to the RoseTTAFold model colored by predicted RMS error ranging from blue for 1.5 Å or less to red 3 Å or greater. The actual Cα-RMSD between the predicted structure and final refined structure is 3.0 Å over the beta-sheets. Figure prepared with ChimeraX (35).

Solution of X-ray structures by molecular replacement (MR) often requires quite accurate models. The much higher accuracy of the RoseTTAFold method than currently available methods prompted us to test whether it could help solve previously unsolved challenging MR problems and improve the solution of borderline cases. Four recent crystallographic datasets (summarized, including resolution limits, in table S2), which had eluded solution by MR using models available in the PDB, were reanalyzed using RoseTTAFold models: glycine N-acyltransferase (GLYAT) from *Bos taurus* (fig. S5A), a bacterial oxidoreductase (fig. S5B), a bacterial surface layer protein (SLP) (Fig. 2A) and the secreted protein Lrbp from the fungus *Phanerochaete chrysosporium* (Fig. 2B and fig. S5C). In all four cases, the predicted models had sufficient structural similarity to the true structures that led to successful MR solutions (see Methods for details; the per-residue error estimates by DeepAccNet (12) allowed the more accurate parts to be weighted more heavily). The increased prediction accuracy was critical for success in all cases, as models made with trRosetta did not yield MR solutions.

To determine why the RoseTTAFold models were successful, where PDB structures had previously failed, we compared the models to the crystal structures we obtained. The images in Fig. 2A and fig. S5 show that in each case, the closest homolog of the known structure was a much poorer model than the RoseTTAFold model; in the case of SLP, only a distant model covering part of the N-terminal domain (38% of the sequence) was available in the PDB, while no homologs of the C-terminal domain of SLP or any portion of Lrbp could be detected using HHsearch (13).

Building atomic models of protein assemblies from cryo-EM maps can be challenging in the absence of homologs with known structures. We used RoseTTAFold to predict the p101 G_βγ_ binding domain (GBD) structure in a heterodimeric PI3K_γ_ complex. The top HHsearch hit has a statistically insignificant E-value of 40 and only covers 14 residues out of 167 residues. The predicted structure could readily fit into the electron density map despite the low local resolution (Fig. 2C, top; trRosetta failed to predict the correct fold with the same MSA input (fig. S6)). The Cα-RMSD between the predicted and the final refined structure is 3.0 Å over the beta-sheets (Fig. 2C, bottom).

## Providing insights into biological function

Experimental structure determination can provide considerable insight into biological function and mechanism. We investigated whether structures generated by RoseTTAFold could similarly provide new insights into function. We focused on two sets of proteins: first, G protein-coupled receptors of currently unknown structure, and second, a set of human proteins implicated in disease. Benchmark tests on GPCR sequences with determined structures showed that RoseTTAFold models for both active and inactive states can be quite accurate even in the absence of close homologs with known structures (and better than those in current GPCR model databases (14, 15); fig. S7) and that the DeepAccNet model quality predictor (12) provides a good measure of actual model accuracy (fig. S7D). We provide RoseTTAFold models and accompanying accuracy predictions for closed and open states of all human GPCRs of currently unknown structure.

Protein structures can provide insight into how mutations in key proteins lead to human disease. We identified human proteins without close homologs of known structure that contain multiple disease-causing mutations or have been the subject of intensive experimental investigation (see Methods). We used RoseTTAFold to generate models for 693 domains from such proteins. Over one-third of these models have a predicted lDDT > 0.8, which corresponded to an average Cα-RMSD of 2.6 Å on CASP14 targets (fig. S8). Here, we focus on three examples that illustrate the different ways in which structure models can provide insight into the function or mechanisms of diseases.

**Fig. 3:**
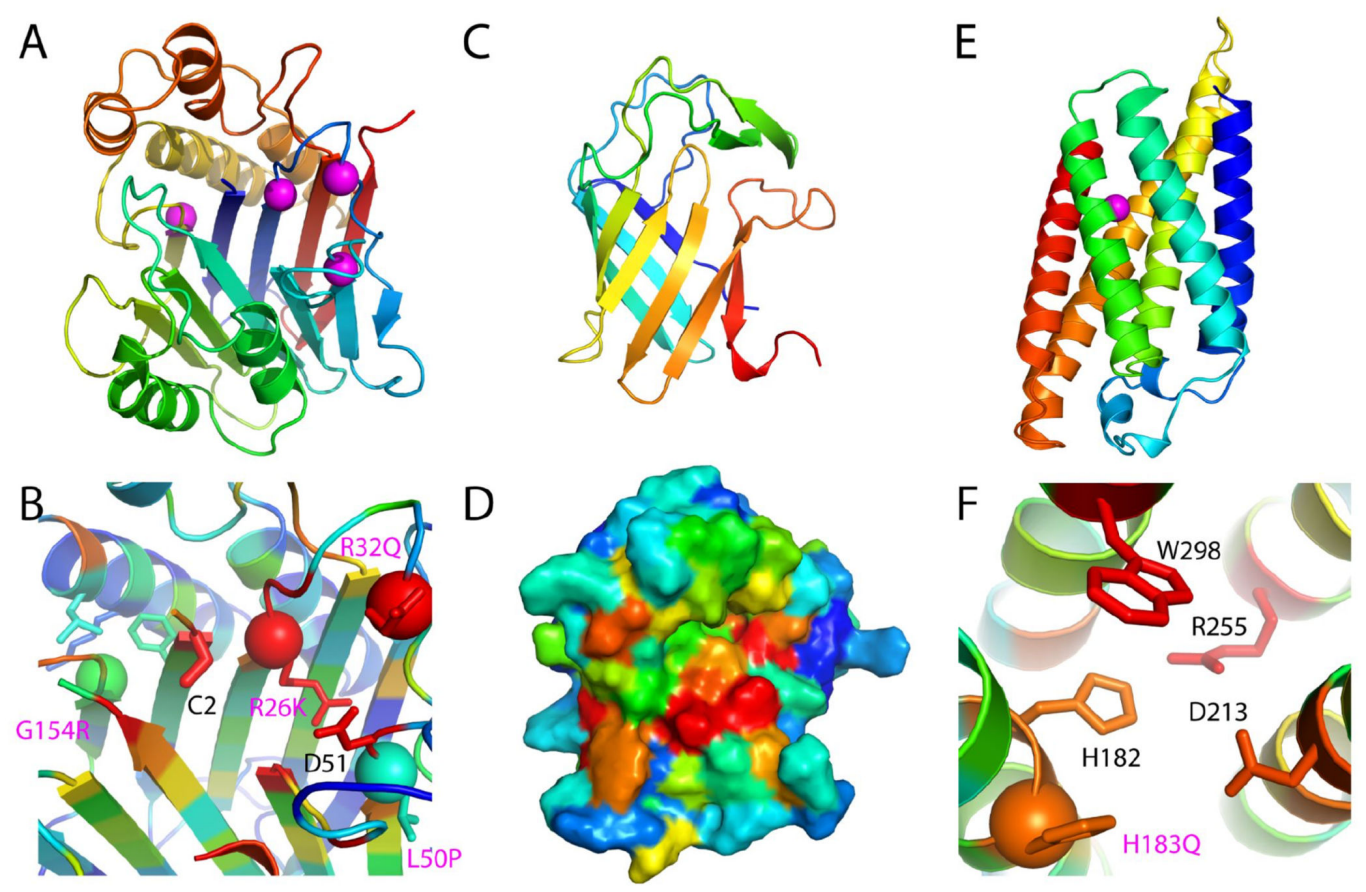
RoseTTAFold models provide insights into function. **(A)** TANGO2 model, colored in a rainbow from the N-terminus (blue) to the C-terminus (red), adopts an Ntn hydrolase fold. Pathogenic mutation sites are in magenta spheres. **(B)** Predicted TANGO2 active site colored by ortholog conservation in rainbow scale from variable (blue) to conserved (red) with conserved residues in stick and labeled. Pathogenic mutations (spheres with wild-type side chains in the sticks) are labeled in magenta; select neighboring residues are depicted in the sticks. **(C)** ADAM33 prodomain adopts a lipocalin-like barrel shown in a rainbow from N-terminus (blue) to C-terminus (red). **(D)** ADAM33 model surface rendering colored by ortholog conservation from blue (variable) to red (conserved), highlighting a conserved surface patch. **(E)** CERS1 transmembrane structure prediction is colored from N-terminus (blue) to C-terminus (red), with a pathogenic mutation in TMH2 near a central cavity in magenta. **(F)** Zoom of CERS1 active site with residues colored by ortholog conservation from variable (blue) to conserved (red). Residues that contribute to catalysis (H182 and D213) or are conserved (W298 and D213) line the cavity. The conserved pathogenic mutation is adjacent to the active site.

Deficiencies in TANGO2 (transport and Golgi organization protein 2) lead to metabolic disorders, and the protein plays an unknown role in Golgi membrane redistribution into the ER (16, 17). The RoseTTAFold model of TANGO2 adopts an N-terminal nucleophile aminohydrolase (Ntn) fold (Fig. 3A) with well-aligned active site residues that are conserved in TANGO2 orthologs (Fig. 3B). Ntn superfamily members with structures similar to the RoseTTAFold model suggest that TANGO2 functions as an enzyme that might hydrolyze a carbon-nitrogen bond in a membrane component (18). Based on the model, known mutations that cause disease (magenta spheres in Fig. 3A) could act by hindering catalysis (R26K, R32Q, and L50P, near active site) or produce steric clashes (G154R) (19) in the hydrophobic core. By comparison, a homology model based on very distant (<15% sequence identity) homologs had multiple alignment shifts that misplace key conserved residues (fig. S9 and table S3)

The ADAM (A Disintegrin And Metalloprotease) and ADAMTS families of metalloproteases are encoded by over 40 human genes, mediate cell-cell and cell-matrix interactions (20, 21) and are involved in a range of human diseases, including cancer metastasis, inflammatory disorders, neurological diseases and asthma (21, 22). The ADAMs contain prodomain and metalloprotease domains; the fold of the metalloprotease is known (23, 24), but not that of the prodomain, which has no homologs of known structure. The RoseTTAFold predicted structure of the ADAM33 prodomain has a lipocalin-like beta-barrel fold (Fig. 3C) belonging to an extended superfamily that includes metalloprotease inhibitors (MPIs) (25). There is a cysteine in an extension following the predicted prodomain barrel; taken together, these data are consistent with experimental data suggesting that the ADAM prodomain inhibits metalloprotease activity using a cysteine switch (26). Conserved residues within ADAM33 orthologs line one side of the barrel and likely interact with the metalloprotease (Fig. 3D).

Transmembrane spanning Ceramide synthase (CERS1) is a key enzyme in sphingolipid metabolism which uses acyl-CoA to generate ceramides with various acyl chain lengths that regulate differentiation, proliferation, and apoptosis (27). Structure information is not available for any of the CerS enzymes or their homologs, and the number and orientation of transmembrane helices (TMH) are not known (28). The RoseTTAFold CERS1 model for residues 98 to 304 (Pfam TLC domain) (29) includes six TMH that traverse the membrane in an up and down arrangement (Fig. 3E). A central crevice extends into the membrane and is lined with residues required for activity (His182 and Asp213) (30) or conserved (W298), as well as a pathogenic mutation (H183Q) found in progressive myoclonus epilepsy and dementia that decreases ceramide levels (31). This active site composition (His182, Asp 213, and potentially a neighboring Ser212) suggests testable reaction mechanisms for the enzyme (Fig. 3F).

## Direct generation of protein-protein complex models

**Fig. 4:**
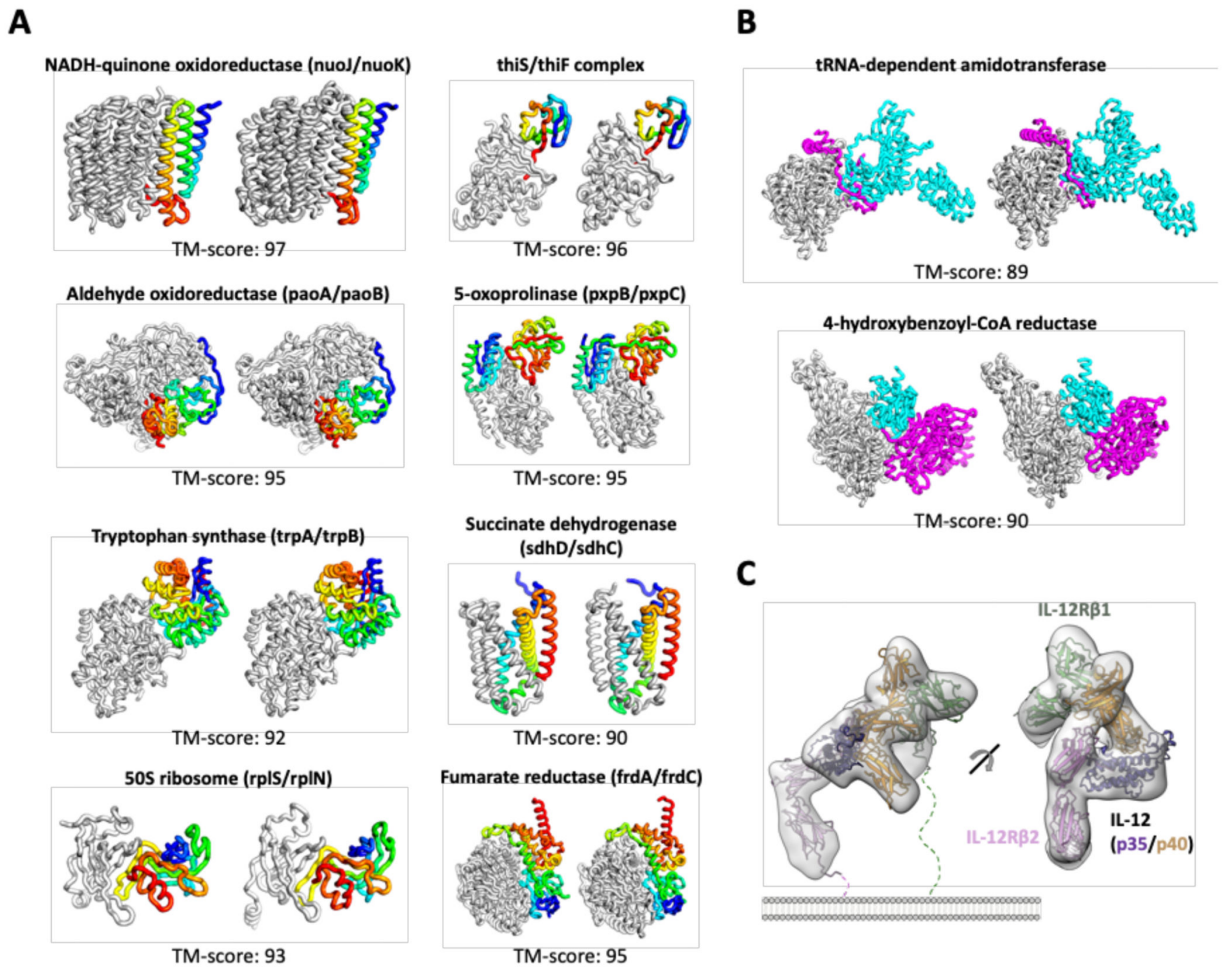
Complex structure prediction using RoseTTAFold. **(A, B)** Prediction of structures of *E.coli* protein complexes from sequence information. Experimentally determined structures are on the left, RoseTTAFold models, on the right; the TMscores below indicate the extent of structural similarity. **(A)** Two chain complexes. The first subunit is colored in gray, and the second subunit is colored in a rainbow from blue (N-terminal) to red (C-terminal). **(B)** Three chain complexes. Subunits are colored in gray, cyan, and magenta. **(C)** IL-12R/IL-12 complex structure generated by RoseTTAFold fits the previously published cryo-EM density (EMD-21645).

The final layer of the end-to-end version of our 3-track network generates 3D structure models by combining features from discontinuous crops of the protein sequence (two segments of the protein with a chain break between them). We reasoned that because the network can seamlessly handle chain breaks, it might be able to predict the structure of protein-protein complexes directly from sequence information. Rather than providing the network the sequence of a single protein, with or without possible template structures, two or more sequences (and possible templates for these) can be input, with the output the backbone coordinates of two or more protein chains. Thus, the network enables the direct building of structure models for protein-protein complexes from sequence information, short circuiting the standard procedure of building models for individual subunits and then carrying out rigid-body docking. In addition to the great reduction in compute time required (complex models are generated from sequence information in ~30 min on a 24G TITAN RTX GPU), this approach implements “flexible backbone” docking almost by construction as the structures of the chains are predicted in the context of each other. We tested the end-to-end 3-track network on paired sequence alignments for complexes of known structures (32) (see Methods and table S4 for details) containing two (Fig. 4A) or three (Fig. 4B) chains, and in many cases, the resulting models were very close to the actual structures (TM-score (33) > 0.8). Information on residue-residue co-evolution between the paired sequences likely contributes to the accuracy of the rigid body placement as more accurate complex structures were generated when more sequences were available (fig. S10). The network was trained on monomeric proteins, not complexes, so there may be some training set bias in the monomer structures, but there is none for the complexes.

To illustrate the application of RoseTTAFold to complexes of unknown structure with more than three chains, we used it to generate models of the complete four-chain human IL-12R/IL-12 complex (Fig. 4C and fig. S11). A previously published cryo-EM map of the IL-12 receptor complex indicated a similar topology to that of the IL-23 receptor; however, the resolution was not sufficient to observe the detailed interaction between IL-12Rβ2 and IL-12p35 (34). Such an understanding is important for dissecting the specific actions of IL-12 and IL-23 and generating inhibitors that block IL-12 without impacting IL-23 signaling. The RoseTTAFold model fits the experimental cryo-EM density well and identified a shared interaction between Y189 in IL-12p35 and G115 in IL-12Rβ2 analogous to the packing between W156 in IL-23p19 with G116 in IL-23R. In addition, the model suggests a role for the IL-12Rβ2 N-terminal peptide (residue 24-31) in IL-12 binding not observed in the IL-12 cryo-electron microscopy (IL-12Rβ2 D26 may interact with nearby K190 and K194 in IL-12p35), which may provide an avenue to target the interaction between IL-12 and IL-12Rβ2 specifically.

## Conclusions

RoseTTAFold enables solutions of challenging X-ray crystallography and cryo-EM modeling problems, provides insight into protein function in the absence of experimentally determined structures, and rapidly generates accurate models of protein-protein complexes. Further training on protein-protein complex datasets will likely further improve the modeling of the structures of multiprotein assemblies. The approach can be readily coupled with existing small molecule and protein binder design methodology to improve computational discovery of new protein and small molecule ligands for targets of interest. The simultaneous processing of sequence, distance, and coordinate information by the three-track architecture opens the door to new approaches incorporating constraints and experimental information at all three levels for problems ranging from cryo-EM structure determination to protein design.

## Supplementary Material

Supplementary Material

## Data Availability

The GPCR models of unknown structures have been deposited to http://files.ipd.uw.edu/pub/RoseTTAFold/all_human_GPCR_unknown_models.tar.gz and http://files.ipd.uw.edu/pub/RoseTTAFold/GPCR_benchmark_one_state_unknown_models.ta r.gz. The model structures for structurally uncharacterized human proteins have been deposited to http://files.ipd.uw.edu/pub/RoseTTAFold/human_prot.tar.gz. The atomic models have been deposited at the Protein Data Bank (PDB) with accession codes PDB: 7MEZ (full PI3K complex structure). The structures for GLYAT, oxidoreductase, SLP, and Lrbp proteins will be deposited in the PDB when final processing is completed. The method is available as a server at https://robetta.bakerlab.org (RoseTTAFold option), and the source code and model parameters are available at https://github.com/RosettaCommons/RoseTTAFold or Zenodo (36).

## One-Sentence Summary

Accurate protein structure modeling enables the rapid solution of protein structures and provides insights into function.
